# Gas embolism under standard versus low pneumoperitoneum pressure during laparoscopic liver resection (GASES): study protocol for a randomized controlled trial

**DOI:** 10.1186/s13063-021-05678-8

**Published:** 2021-11-15

**Authors:** Danfeng Jin, Mingyue Liu, Jian Huang, Yongfeng Xu, Luping Liu, Changhong Miao, Jing Zhong

**Affiliations:** 1 Department of Anesthesiology, Zhongshan Hospital Fudan University, Shanghai, China; 2 Department of Hepatology, Zhongshan Hospital Fudan University, Shanghai, 200032 China; 3 Department of Anesthesiology, Zhongshan Wusong Hospital Affiliated to Fudan University, Shanghai, 200940 China; 4 Fudan Zhangjiang Institute, Shanghai, 201203 China

**Keywords:** Gas embolism, Laparoscopic liver resection (LLR), Pneumoperitoneum pressure (PP), Inferior vena cava-collapsibility index (IVC-CI), Randomized controlled trial

## Abstract

**Background:**

Gas embolism induced by CO_2_ pneumoperitoneum is commonly identified as a risk factor for morbidity, especially cardiopulmonary morbidity, after laparoscopic liver resection (LLR) in adults. Increasing pneumoperitoneum pressure (PP) contributes to gas accumulation following laparoscopy. However, few studies have examined the effects of PP in the context of LLR. In LLR, the PP-central venous pressure (CVP) gradient is increased due to hepatic vein rupture, hepatic sinusoid exposure, and low CVP management, which together increase the risk of CO_2_ embolization. The aim of this study is to primarily determine the role of low PP (10 mmHg) on the incidence of severe gas embolism.

**Methods:**

Adult participants (*n* = 140) undergoing elective LLR will be allocated to either a standard (15 mmHg) or low (10 mmHg) PP group. Anesthesia management, postoperative care, and other processes will be performed similarly in both groups. The occurrence of severe gas embolism, which is defined as gas embolism ≥ grade 3 according to the Schmandra microbubble method, will be detected by transesophageal echocardiography (TEE) and recorded as the primary outcome. The subjects will be followed up until discharge and followed up by telephone 1 and 3 months after surgery. Postoperative outcomes, such as the Post-Operative Quality of Recovery Scale, pain severity, and adverse events, will be assessed. Serum cardiac markers and inflammatory factors will also be assessed during the study period. The correlation between intraoperative inferior vena cava-collapsibility index (IVC-CI) under TEE and central venous pressure (CVP) will also be explored.

**Discussion:**

This study is the first prospective randomized clinical trial to determine the effect of low versus standard PP on gas embolism using TEE during elective LLR. These findings will provide scientific and clinical evidence of the role of PP.

**Trial status:**

Protocol version: version 1 of 21-08-2020

**Trial registration:**

ChiCTR2000036396 (http://www.chictr.org.cn). Registered on 22 August 2020.

**Supplementary Information:**

The online version contains supplementary material available at 10.1186/s13063-021-05678-8.

## Background

Laparoscopy is a common surgical treatment for various hepatic diseases, largely due to the enhanced recovery after surgery [1]. CO_2_ pneumoperitoneum is necessary for laparoscopy to achieve sufficient operative space. Pneumoperitoneum-related gas embolism is a serious complication in patients who undergo laparoscopic liver resection (LLR), which increases the risk of mortality and postoperative complications [2, 3]. Multiple factors, including pneumoperitoneum pressure (PP), affect gas accumulation at both the tissue and cellular levels.

Currently, the PP range of about 15 mmHg is commonly used in LLR. Increasing the PP can change the surgical peritoneal environment and lead to peritoneal dissemination and hypoxia [4–6], which may eventually cause respiratory and circulatory injury [7]. In LLR, the PP-central venous pressure (CVP) gradient is enlarged due to hepatic vein rupture [8], hepatic sinusoid exposure, and low CVP management, all of which increase the risk of CO_2_ embolization [9]. Therefore, a low level of 6–10 mmHg PP is recommended in laparoscopic surgery, according to recent studies [10, 11]. A low PP has been shown to improve postoperative pain and may also decrease liver and kidney injuries [12]. The link between intraperitoneal pressure and embolization has been demonstrated in experimental animal models [13]. However, the effect of PP level on gas embolism during LLR has not been comprehensively delineated among patients.

Transesophageal echocardiography (TEE) is invaluable in patients with cardioembolic events because of its high sensitivity and specificity for defining the detailed structure and function of the cardiovascular system [14]. In addition, there is a strong correlation between the TEE-derived inferior vena cava (IVC) diameter and the CVP [15]. The IVC collapsibility index (IVC-CI) is inversely correlated with CVP [16], but this correlation may be invalid in LLR when a high PP is added [17].

### Objectives

The aim of the present study was to assess the effects of standard (15 mmHg) versus low PP (10 mmHg) on the occurrence and severity of gas embolism using TEE during elective LLR, to evaluate the corresponding postoperative recovery with the Post-Operative Quality of Recovery Scale (PQRS) [18], and attempt to explore the correlation between IVC-CI and CVP on postoperative outcomes.

### Trial design

**Fig. 1:**
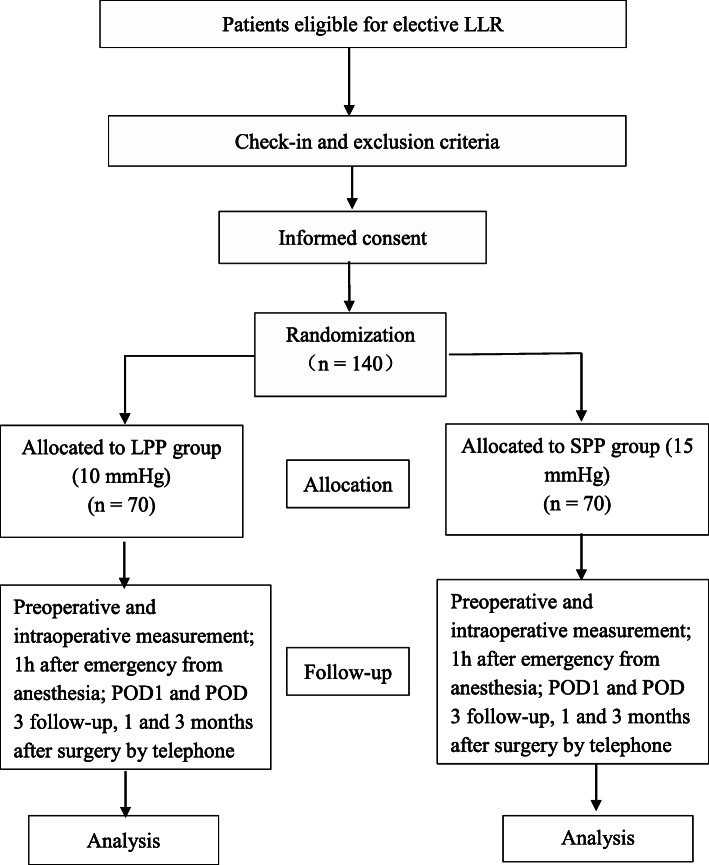
CONSORT flowchart illustrating the randomization and flow of study participants. LPP, low pneumoperitoneum pressure; SPP, standard pneumoperitoneum pressure; POD, postoperative day

GASES is a prospective, randomized, controlled, assessor-blinded, two-arm trial initiated by investigators. The contents of this protocol are based on the recommendations for interventional trials (SPIRIT) guidelines [19]. The SPIRIT checklist is included in Additional file 1. In total, 140 patients will be randomly assigned to one of two different PP (CONSORT diagram, Fig. 1).

The GASES trial tests the hypothesis that, in patients undergoing laparoscopic liver surgery, low PP (10 mmHg), compared to standard PP (15 mmHg), reduces the incidence of severe gas embolism, which will be recorded as the primary outcome. Severe gas embolism is defined as gas embolism ≥grade 3 in the right atrioventricular system from the four-chamber cardiac plane of the middle esophagus, as detected by TEE (GE VENUE R2) during the LLR according to the Schmandra microbubble method [20].

## Methods/design

The GASES trial will be conducted during laparoscopic hepatectomy (patients diagnosed with hepatic tumor, hemangioma, and hepatic cyst) at Zhongshan Hospital Fudan University, Shanghai, China.

### Selection of participants

Patients will be included in the GASES trial if they comply with the inclusion and exclusion criteria outlined below:

### Inclusion criteria

For inclusion, adult patients must meet all the following criteria:

1. Patients scheduled to perform elective LLR under general anesthesia
2. 18–75 years old
3. Body mass index (BMI) between 18.5 kg/m^2^ and 30 kg/m^2^
4. From whom written informed consent is obtainable either from the patient or from a legal representative

### Exclusion criteria

Patients will be excluded for any of the following reasons:

1. Patient with acute heart failure
2. Patient with acute coronary insufficiency
3. Patient with severe renal failure (defined as creatinine clearance < 30 mL/min or requiring renal replacement therapy)
4. Patient with preoperative shock (defined as the need for vasoactive drugs before surgery)
5. History of neuromuscular disease and abdominal cancer with adhesion
6. Contradiction to use TEE because of severe esophageal varices
7. Participation in other drug trials in the 30 days prior to enrollment
8. Parturient or breast-feeding women
9. Inability to express oneself correctly, poor compliance, and inability to complete the test according to the study plan
10. Patient’s or relative’s refusal to participate

### Randomization and blinding

Randomization will be conducted over a dedicated, password-protected, SSL-encrypted website (edc.fudan.edu.cn) to allow immediate and concealed allocation. Each patient will be given a patient number and randomization number. The allocation sequence, with an allocation ratio of 1:1 for each group, will be generated using a minimization algorithm stratified according to risk of intraoperative exposure of the hepatic vein, liver cirrhosis status, and history of abdominal surgery. Participant allocation will be performed by a specific local investigator; the investigator will log into the randomization system using a personal ID code and will enter any relevant information. This will ensure that the patient receives only surgery under the randomized PP. The process of sequence generation and storage will be managed by an independent database manager who is not involved in patient care.

Surgeons and assessors of intraoperative gas embolism grades and PQRS [21] will be blinded to the treatment allocation. At least two assessors will be involved in the study. One assessor will determine the intraoperative gas embolism grades, and the second assessor will perform postoperative visits and assessments of the primary and secondary endpoints. All patients will be followed up by researchers who are blinded to the intraoperative grouping, and each patient will be evaluated by the same researcher during the perioperative period.

### Trial interventions

All included patients will fast for 6–8 h before surgery and will be allocated to one of the following two study groups:

1. Low pneumoperitoneum pressure (LPP) group: 10 mmHg PP will be set during surgery.
2. Standard pneumoperitoneum pressure (SPP) group: 15 mmHg PP will be set during surgery.

After anesthesia induction, the TEE probe will be gently inserted into the esophagus to assess the incidence and grades of gas embolism until the end of the surgery. Infusion will be performed through the peripheral venous line rather than the central venous catheter in order to reduce interference with the evaluation of gas embolism. The PP will be set at 10 or 15 mmHg throughout the operation, according to the group allocation. The patients will receive deep neuromuscular blockade with cisatracurium throughout surgery to maintain a train of four (TOF) = 0 and post-tetanic count (PTC) ≥ 1 to satisfy the operation space. To avoid the confounding factor of surgical skills, all surgeries will be performed by a surgeon for two groups and all patients. Neuromuscular blockade pharmacological reversion will be achieved with neostigmine (2.5 mg or 30–50 μg/kg), according to usual care.

### Standard procedures

To avoid interference with the trial intervention, routine elements of perioperative anesthesia care (including general anesthesia, postoperative pain management, physiotherapeutic procedures, and fluid management) will be performed according to the clinical routine of Zhongshan Hospital Fudan University. The following approaches are suggested (not mandatory) for anesthetic management:

1. Low CVP anesthesia will be administered during the operation. The CVP will be encouraged to be maintained at 5 ± 2 mmHg. Dobutamine (1–3 μg/kg/min) and nitroglycerin (5 μg/min) can be used to achieve a low CVP if necessary.
2. Restrictive fluid therapy will be used to reduce the increase of intraoperative CVP until the liver mass is removed.
3. Patients in both groups will be ventilated in a volume-controlled ventilation mode. The tidal volume (VT) will be set at 8 mL/kg predicted body weight (PBW), without intraoperative high positive end-expiratory pressure (PEEP). The respiratory rate will be set at 10–12 bpm, and the inspiratory to expiratory time (I:E) ratio will be 1:2.
4. Preoperative invasive arterial blood pressure monitoring will be established for circulation management and perioperative blood gas analysis. The mean arterial pressure will be maintained at ≥ 60 mmHg.
5. Cerebral oxygen and cardiac output are monitored during the perioperative period.
6. Blood products will be given to maintain hemoglobin at a level > 8 g/dl (in patients with no history of ischemic heart disease or ≥ 10 g/dl otherwise).
7. Normothermia and normoglycemia will be maintained throughout the surgical period.
8. Appropriate prophylactic antibiotics will be used as recommended.
9. Oxygenation (pulse oximetry) will be maintained at ≥ 94%. If hypoxemia, defined as peripheral oxygen saturation (SpO_2_) < 90% occurs for > 1 min, FiO_2_ will be increased in steps of 0.1 until 1.0, and recruitment maneuvers (RM) will be applied. If hypercapnia (P_ET_CO_2_ > 60 mmHg) with respiratory acidosis (pHa < 7.20) occurs, the respiratory rate will be increased (maximum 30/min), and VT will be increased stepwise up to 10 mL/kg PBW.

Other procedures will follow the Safe Surgery Checklist of the World Health Organization as published (www.who.int/patientsafety/safesurgery/en/index.html).

### Outcomes

The primary outcome will be the incidence of gas embolism ≥ grade 3 in the right atrioventricular system from the four-chamber cardiac plane of the middle esophagus; this will be detected by TEE (GE VENUE R2) over the duration of the LLR. The grades of gas embolism will be determined according to the Schmandra microbubble method as follows:

Grade 0: No bubbles observed in the right ventricular system

Grade 1: A single bubble observed in right atrium (RA), right ventricle (RV), or right ventricular outflow tract (RVOT).

Grade 2: Multiple bubbles observed, but only occupying  part of the RA, RV, or RVOT.

Grade 3: Multiple bubbles observed, occupying most of the RA, RV, or RVOT.

Grade 4: Bubbles occupying the whole RA, RV, or RVOT.

The secondary outcome measures will be as follows:

1. PQRS
2. Correlations between intraoperative IVC-CI and CVP. IVC-CI will be defined as (IVC_max_ − IVC_min_)/IVC_max_, which is the difference between the maximum inspiratory diameter and minimum expiratory diameter divided by the maximum inspiratory diameter in intubated patients with mechanical ventilation during LLR.
3. Intra-operative complications (hypotension, hypothermia, hypoxia) related to gas embolism
4. Postoperative organ dysfunction (Appendix 1)
5. Postoperative complications within 30 days
6. Need for unexpected intensive care unit (ICU) admission or readmission
7. Number of hospital-free days at day 28
8. 90-day survival
9. In-hospital survival
10. Satisfaction of surgeon

Blood samples will be collected preoperatively, in postanesthesia care unit (PACU), and on postoperative day (POD) 1. Samples will be analyzed centrally for CRP, hs-CRP, cTnT, NT-proBNP, and IL-1, IL-6, IL-8, and IL-10. The standard operating procedure for collecting and processing blood samples is available in Additional file 2.

### Quality of recovery

The Chinese version of the PQRS status test will be used to assess the quality of recovery in patients undergoing LLR (Additional file 3). The PQRS is a validated multidimensional patient report outcome tool [18, 21, 22] designed to assess patients’ recovery to baseline status in the postoperative period (www.postopqrs.com). In every patient, a baseline measurement of PQRS will be performed prior to surgery. After surgery, the measurement of the PQRS will be repeated 40 min after arrival in the post-anesthesia care unit (PACU), as well as in the ward on the morning of POD 1 and POD 3. The PQRS is a verbal survey tool that depicts recovery in the following five domains: physiologic, nociceptive, emotive, functional, and cognitive, and also collects the overall patient perspective. Each of these domains is assessed with multiple items on an ordinal scale and is compared to the baseline to evaluate recovery. Recovery is a dichotomized outcome defined by a return to baseline values or better at each postoperative measurement timepoint. Overall recovery requires recovery in all assessed domains, and failure in any of the domains results in failure of overall recovery.

### Study visits and data collection

**Fig. 2:**
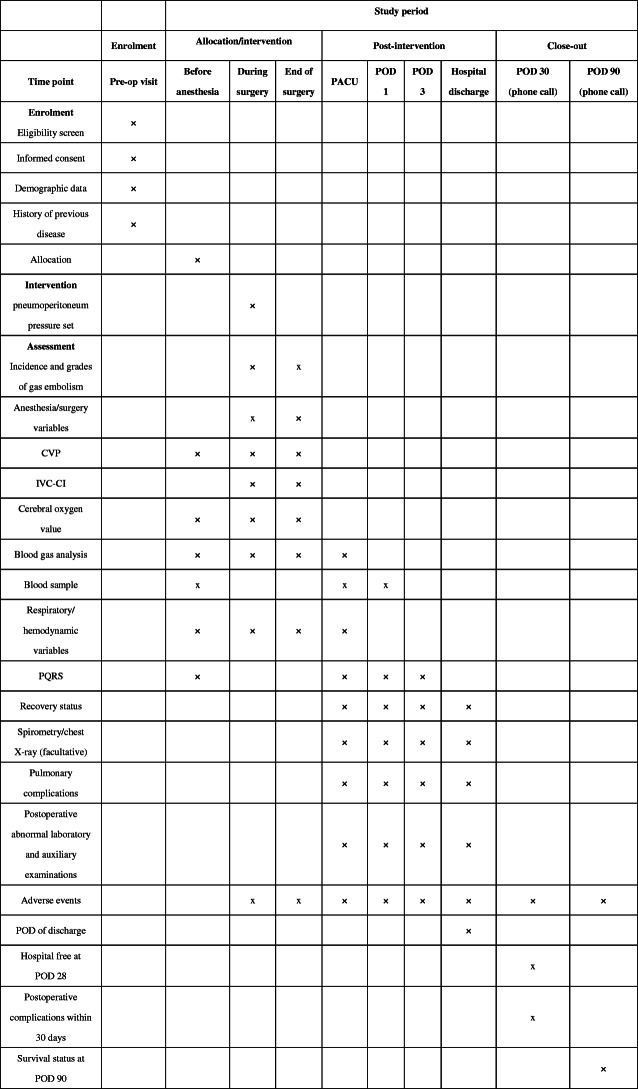
Schedule of enrolment, interventions, and assessments. Pre-op, pre-operative; PACU, post-anesthesia care unit; POD, postoperative day; CVP, central venous pressure; IVC-CI, inferior vena cava-collapsibility index; PQRS, Post-Operative Quality of Recovery Scale

Patients will be visited preoperatively, intraoperatively, daily between POD 1 and 3, and on discharge. Patients will be contacted by phone on PODs 30 and 90 (Fig. 2).

The patients will be screened according to the inclusion criteria. All patients meeting the inclusion criteria will be registered in a screening log file. The investigators will explain the course and purpose of the study and provide written information on the study. Patients willing to participate in the study will be required to provide written informed consent (Additional file 2).

Data will be registered in both the paper case report form (CRF) and the electronic case report form (eCRF) on electronic data capture (EDC) system by trial or clinical personnel under the supervision of the trial investigators. In the case of inconsistency, the paper CRF will be used as a gold standard.

The following data will be collected:

Pre-randomization and baseline characteristics: Demographic data, including age, height, weight, sex, and BMI; surgical characteristics, including preoperative diagnosis, and type, location, number, and size of the primary liver tumor; co-morbidities, including coronary heart disease Y/N and Canadian Cardiovascular Society (CCS) score if Y, chronic heart failure Y/N and New York Heart Disease Association (NYHA) score if Y, atrial flutter or fibrillation Y/N, premature systole Y/N, conduction block Y/N, pulmonary ventilation dysfunction Y/N, chronic obstructive pulmonary disease Y/N and specific therapy if Y, hypertension Y/N, diabetes mellitus Y/N, chronic alcoholism Y/N, obstructive sleep apnea-hypopnea syndrome Y/N, and active smoking Y/N; American Society of Anesthesiologists (ASA) physical status; preoperative use of statins Y/N; preoperative use of aspirin Y/N; biological indices, including CRP, hs-CRP, c-TnT, NT-proBNP, IL-1, IL-6, IL-8, and IL-10; values of blood routine examination, hepatic and renal function, and coagulation function; measurements at baseline in the operation room (without oxygenation) before anesthesia induction, including measurements of heart rate, respiratory rate, CVP, SpO_2_, non-invasive blood pressure (NIBP), invasive blood pressure (IBP), artery blood gas (ABG) analysis and cerebral oxygen saturation (PQRS-0 is measured pre-operatively as the baseline).

At randomization (stratification): risk of intraoperative exposure of hepatic vein Y/N, liver cirrhosis status Y/N, and history of abdominal surgery Y/N.

During the surgical procedure, the TEE probe will be placed through the esophagus until extubation, and the following data will be collected:

1. Anesthetic data: type and doses of hypnotics, opioids, and muscle relaxants; duration of anesthesia; total volume of maintenance fluid; total number of blood products; ventilator settings (VT, respiratory rate, PEEP, FiO_2_); baseline (and then hourly) values for P_ET_CO_2_; cerebral oxygen monitoring values; blood pressure (systolic, diastolic, and mean); cardiac index, stroke volume variation, and cardiac performance index measured by the PULSION medical system (not mandatory); infusion rate of vasoactive drugs (dobutamine, ephedrine hydrochloride, noradrenaline, and other), and hourly urine output.
2. Surgical data: type of surgery, duration of surgery, total volume of blood loss, surgical complications Y/N, and surgeon satisfaction.
3. Intraoperative TEE monitoring data: intraoperative gas embolism grade, baseline for IVC-CI, and eccentricity index (EI) after anesthesia induction, and the grades and duration during gas embolism occurrence. EI is used to evaluate right ventricular volume and pressure overload [23]. IVC-CI and EI will also be recorded at the time of trocar insertion, the beginning of hepatic parenchymal transection, hepatic tumor removal, and following completion of the surgery. Vital signs, CVP, P_ET_CO_2_, cerebral oxygen monitoring values, ABG, and IVC-CI will also be recorded when any grade of gas embolism is detected by TEE.

After the surgical procedure, the following indices will be collected before hospital discharge:

1. Postoperative care pathway (surgical ward Y/N and ICU Y/N)
2. Daily lowest values for heart rate, blood pressure, peripheral O_2_ saturation, respiratory rate, and temperature
3. Plasma ALT, bilirubin, creatinine, lactate, CRP, IL-1, IL-6, IL-8, IL-10, NT-proBNP, and cTnT (standard laboratory values, if any)
4. PQRS-1 (PACU), PQRS-2 (POD 1) and PQRS-3 (POD 3)
5. Postoperative complications (Y/N, type, and date of diagnosis), including intra-abdominal hemorrhage, ascites, bile leakage, intra-abdominal abscess, and post-resection liver failure
6. Postoperative pulmonary complications (Appendix 1), as assessed by finger pulse oxygen saturation, blood gas analysis (if any), and chest X-ray (if any)
7. Unexpected ICU admission Y/N
8. Length of stay in the ICU and surgical ward
9. Date of hospital discharge
10. Death (Y/N and date)

Thirty days after surgery:

1. Postoperative complications (Y/N, type, and date of diagnosis)
2. Survival status (If the patient is deceased, date of death)

Ninety days after surgery:

1. Survival status (if the patient is deceased, date of death)

### Study dropouts

Participation in the trial is voluntary, and patients will have the right to withdraw consent at any time, for any reason, without any consequence for further medical treatment. The reasons and circumstances for study discontinuation will be documented in the CRF. The patients’ participation in this study can also be ended by the investigator if the patient is uncooperative and/or does not attend the study visits. In this case, patient data collected up until the point of removal will be included in the analysis; if too many data are missing (e.g., missing baseline, study visits, and other measurements included in the primary outcome), the patient will be replaced by a new patient. Patients who convert to open surgery will be treated according to the standard of care, based on the findings during surgery. These patients will be excluded from the study, and patient data until this moment will be included in the analysis.

### Handling of data

Two members of the research team will perform study monitoring. Remote monitoring will be performed to signal early aberrant patterns, issues with consistency, credibility, and other anomalies. Patient data will be collected in pseudonymous form using a patient (identification) number composed of three digits corresponding to the patient inclusion number. Study data will be collected and managed using an investigator-initiated trial EDC (IIT-EDC) tools. IIT-EDC is a password-protected, intranet-based application designed to support data records for research studies (website: http://10.15.7.137). Full access to the final trial dataset will be granted to selected investigators. All original records, including consent forms, reports of suspected unexpected serious adverse events (SAE), and relevant correspondences, will be archived at the trial sites for 10 years. The clean trial database file will be anonymized and maintained for 10 years.

### Plans for communicating important protocol amendments to relevant parties (e.g., trial participants and ethical committees)

A “substantial amendment” is defined as an amendment to the protocol or any other supporting documentation that is likely to affect to a significant degree one or more of the following: the safety or physical or mental integrity of the subjects of the trial; the scientific value of the trial; the conduct or management of the trial; or the quality or safety of any intervention used in the trial.

All substantial amendments will be notified to the Institutional Review Board of Zhongshan Hospital Fudan University (China) and to the competent authorities. Non-substantial amendments will be recorded and filed. In the case that amendments concern or affect participants in any way, they will be informed about the changes. If required, additional consent will be requested and registered. In addition, the online trial registry will be updated accordingly.

### Safety

All adverse events (AE) thought to be related to the trial will be reported to the trial coordinating center. According to local laws and regulations, the participants may receive free treatment provided by our unit or be compensated if any injury related to this study does occur, and all suspected unexpected SAEs will be reported to the data monitoring and safety committee (DMSC). The DMSC is independent of the trial investigators and will perform an ongoing review of safety parameters and overall study conduct. The DMSC comprises two independent experts in large-scale clinical trials and one independent statistician.

The DMSC will be responsible for safeguarding the interests of the trial participants, assessing the safety and efficacy of the interventions during the trial, and monitoring the overall conduct of the clinical trial. To enhance the integrity of the trial, the DMSC may also formulate recommendations relating to the recruitment/retention of participants, their management, improving adherence to protocol-specified regimens and retention of participants, and procedures for data management and quality control.

### Statistical analysis

#### Sample size

The incidence of severe gas embolism in the right ventricular system detected by TEE will be used as the primary outcome parameter. Based on results from preliminary trial, the incidence of grade 3 and above gas embolism in LLR using low and standard intra-abdominal pressure was 45% and 69%, respectively. Sample size was calculated by a web-based calculator (http://powerandsamplesize.com/Calculators/). For the power of 80% and a level of significance of 5% against the two-sided alternative hypothesis, 126 participants are required to detect the observed difference in gas embolism incidence using chi-square test. Expecting 10% drop-out of participants, a sample size of 140 is required.

#### Analysis

Continuous distribution of the data was assessed by visual inspection of histograms and D’Agostino–Pearson’s normality tests. For both arms, the baseline characteristics will be expressed as counts and percentages, means and standard deviations, or medians and interquartile ranges, as appropriate.

Ventilatory parameters and vital signs during the surgery will be analyzed using a mixed-effect model with repeated measures, and with patients as a random effect. No or minimal losses to follow-up for the primary and secondary outcomes are anticipated. The intention-to-treat (ITT, all randomly assigned cases) population is used for all outcomes analysis. The supportive analysis is based on a full analysis set (FAS, all randomly assigned patients who received corresponding PP until liver resection). However, if more than 1% of missing data are found for the primary outcome, a sensitivity analysis using multiple imputations and estimating equation methods will be carried out.

Hypothesis tests will be two-sided with a significance level of 5%, with the exception of the primary outcome.

#### Primary outcome

The effects of the intervention on the incidence of severe gas embolism in the right ventricular system detected by TEE will be reported as numbers and percentages estimated with risk ratios and 95% confidence intervals (CI), as calculated by Wald’s likelihood ratio approximation test and with *χ*^2^ tests for hypothesis testing.

#### Secondary outcomes

Postoperative organ dysfunction and complications within 30 days will be classified according to the classification described by Dindo et al. [24] and defined as a dichotomous composite endpoint, while ICU admission and readmission will be given as a percentage. Scores for PQRS will be given as the mean and standard deviation per time point per treatment arm. The correlation between IVC-CI and CVP will be tested using the correlation analyses.

The effect of the intervention on secondary binary outcomes will be assessed using the risk ratio and 95% CI calculated with Wald’s likelihood ratio approximation test and *χ*^2^ tests for hypothesis testing. The effects of the intervention on hospital-free days at day 28 will be estimated with a Student’s *t* test and reported as the mean difference between the two groups. The consistency of the findings of the Student’s *t* test for the hospital-free days at day 28 will be confirmed according to the mean ratio, as calculated by a generalized additive model considering a zero-inflated beta distribution.

Finally, 90-day mortality will be assessed using Kaplan–Meier curves, and hazard ratios with 95% confidence intervals will be calculated using Cox proportional hazard models without adjustment for covariates. The proportional hazard assumptions will be tested using scaled Schoenfeld residuals, and alternative parametric survival models will be used if the proportionality assumption is not sustained.

#### Subgroup analyses

Treatment effects on the incidence of gas embolism will be analyzed according to the following subgroups: (1) plan of exposing the hepatic vein versus without planning, (2) history versus without history of abdominal surgery, and (3) with versus without liver cirrhosis. The effects on subgroups will be evaluated according to the interaction effects between each subgroup and the study arms using generalized regression models and presented in a forest plot.

Per-protocol analyses: The per-protocol population will consist of patients who truly operate with the pre-specified protocol. Thus, patients will be excluded from this population if they receive PP < 15 mmHg in the standard PP group or PP > 10 mmHg in the low PP group, in any measurement during the surgery.

### Cleaning and locking of the database

The datasets used and/or analyzed during the current study will be made available from the corresponding author upon reasonable request. The database will be locked immediately after all data are entered and all discrepant or missing data are resolved, or, alternatively, if all efforts have been employed and we consider that the remaining issues cannot be fixed. In this step, the data will be reviewed before database locking. The study database will then be locked and exported for statistical analysis. At this stage, permission for access to the database will be removed for all investigators, and the database will be archived.

### Dissemination plans

The results of this research will be disclosed in international peer-reviewed journals. Both positive and negative results will be reported. Patients will receive a laymen summary of the results if they opted-in to receive outcomes at the study level.

### Trial organization

The trial is managed by a team consisting of the chief investigator (Jing Zhong), the trial coordinator (Mingyue Liu), statisticians, the informatics technician responsible for the web-based electronic data capture system (Jian Huang), and independent monitors. A steering committee contributed to the design and revision of the study and will be responsible for the interpretation of data and compilation of the resulting manuscript.

Patient data and safety will be closely monitored by the DMSC. All AEs entered into the eCRF within pre-specified time frames, including severe AEs and suspected unexpected severe adverse reactions, will be monitored by an AE manager (Danfeng Jin), who will provide the DMSC with reports for review.

The coordinator will be responsible for administration and the assistance during trial management and data collection.

## Discussion

This study is the first prospective randomized clinical trial to determine the effect of low PP versus standard PP on gas embolism using TEE during elective LLR. The findings from this study will provide scientific and clinical evidence of the role of PP.

Pneumoperitoneum is closely related to the formation of an air embolism intraoperatively. The overall incidence of intraoperative venous gas embolization was 38% in a study of robot-assisted laparoscopic radical prostatectomy [25] and CO_2_ embolization was observed in 69% of patients who underwent laparoscopic cholecystectomy [26].

LLR was first described in 1995, but developed more slowly than other laparoscopic procedures due to gas embolism and uncontrolled bleeding [27, 28]. An intra-abdominal pressure of 12–15 mmHg of CO_2_ is higher than the normal portal blood pressure of 6–10 mmHg and is therefore capable of reducing portal blood flow and alterations in hepatic function. Although the PP range of about 15 mmHg is routinely used, LLR may be correlated to a higher incidence of air accumulation with surgery of the portal system [29]. It has been reported that gas embolism occurred in 5/7 (71%) of animals that underwent whole laparoscopic hepatectomy, 3/7 (42%) of which experienced a series of arrhythmias correlated with gas embolism [30]. Moreover, venous CO_2_ embolization was detected in all 50 patients who underwent laparoscopic hepatectomy [31].

Complications related to gas embolism should be considered carefully, as gas embolism resulting from pneumoperitoneum establishment has been shown to be associated with a 28% mortality rate after laparoscopic cholecystectomy [32]. CO_2_ gas embolism in laparoscopic surgery may also cause postoperative cardiac arrest [2], cerebral infarction [3], and even death [33]. We reported a case of CO_2_ embolism during laparoscopic nephrectomy without evidence of right-to-left shunt, in which the patient suffered from slurred speech and developed epileptiform seizures 9 h after the surgery [34]. Once gas embolism occurs during LLR, it may affect cardiopulmonary function and even cause neurologic deficits, resulting in delayed postoperative recovery. In this study, TEE will be used for gas embolism monitoring because it is capable of detecting 0.02 mL/kg of gas. Cerebral oxygen will also be observed to prevent cerebral embolism caused by micro emboli. TEE has no effect on cerebral oxygen because the baseline values are mainly related to cardiac function [35].

The occurrence of CO_2_ embolism likely depends on several variables. Surgical duration and size of parenchymal transection are likely to be directly proportional to the risk of gas embolism [36]. Intraperitoneal pressure may also play a role. Indeed, a higher PP led to higher degrees of embolization after laparoscopic IVC injuries in anesthetized pigs [13]. Hence, low-PP surgery is attractive, especially in reducing the incidence of embolism and its related complications. In addition, clinically, the most important benefit of low-PP is lower postoperative pain scores, which accelerate postoperative recovery [12]. No previous studies have compared the incidence of gas thrombosis in low versus standard PP during elective LLR. Therefore, we hypothesized that a low PP (10 mmHg) with controlled-low-CVP will lead to less severe gas embolism and promote postoperative recovery as compared to standard PP (15 mmHg) in elective LLR. To better evaluate the prognosis, PQRS will be used to assess recovery after LLR according to six dimensions of health, including physiologic, nociceptive, emotive, activities of daily living, cognitive, and overall patient perspective [18].

This study also aims to determine the correlation between IVC-CI and CVP on postoperative outcomes. By restrictive fluid therapy, low CVP is achieved in LLR with the benefit of a clear surgical field and reduced blood loss [37]. Low airway pressure (AWP) also reduces bleeding from the hepatic vein [38]. Moreover, it is thought that the risk of pulmonary gas embolism decreases with low AWP when PP is lower than CVP [38]. For the purpose of low AWP, the ventilation tidal volume is set at 8 mL/kg without high PEEP. The relationship between low CVP and gas embolism remains controversial at present [13, 39]; however, there is a theoretic increased risk of CO_2_ embolism at insufflation pressures exceeding the CVP due to the presence of a pressure gradient in the venous circulation [8]. Positive PP can further accentuate this gradient by altering the intrahepatic hemodynamics, which are not reflected in the CVP [39]. Therefore, we compared the effects of IVC-CI and CVP based on different PPs.

In summary, this study will assess the feasibility and effects of low PP (10 mmHg) during LLR. Given the unavoidable air accumulation in laparoscopic surgery, along with a deficiency in scientific and clinical evidence of the efficacy of low PP, this study will provide useful information on this intraoperative management strategy.

## Trial status

Protocol version 1 (21.08.2020). Enrollment of patients started in October 2020. Recruitment is scheduled to be completed on 30.09.2023.

## Supplementary Information

**Additional file 1.**

**Additional file 2.**

**Additional file 3.**

**Additional file 4.**

**Additional file 5: Appendix 1**. Postoperative organ function

## Data Availability

Study data will be collected and managed using an intranet-based, IIT-EDC tools. (website: http://10.15.7.137). Full access to the final trial dataset will be granted to selected investigators.

## Abbreviations

LLR: Laparoscopic liver resection
PP: Pneumoperitoneum pressure
IVC: Inferior vena cava
CI: Collapsibility index
CVP: Central venous pressure
TEE: Transesophageal echocardiography
PQRS: Post-Operative Quality of Recovery Scale
BMI: Body mass index
TOF: Train of four
PTC: Post-tetanic count
VT: Tidal volume
PBW: Predicted body weight
PEEP: Positive end-expiratory pressure
RA: Right atrium
RV: Right ventricle
RVOT: Right ventricular outflow tract
ICU: Intensive care unit
POD: Postoperative day
CRF: Case report form
EDC: Electronic data capture
CCS: Canadian Cardiovascular Society
NYHA: New York Heart Disease Association
ASA: American Society of Anesthesiologists
NIBP: Non-invasive blood pressure
IBP: Invasive blood pressure
ABG: Artery blood gas
EI: Eccentricity index
PACU: Post-anesthesia care unit
CI: Confidence interval
AE: Adverse event
SAE: Serious adverse event
DMSC: The Data Monitoring and Safety Committee
AWP: Airway pressure

## References

[1] Abu Hilal M, Di Fabio F, Teng MJ, Lykoudis P, Primrose JN, Pearce NW (2011). Single-centre comparative study of laparoscopic versus open right hepatectomy. J Gastrointest Surg Off J Soc Surg Aliment Tract.

[2] Kim I-S, Jung J-W, Shin K-M (2012). Cardiac arrest associated with carbon dioxide gas embolism during laparoscopic surgery for colorectal cancer and liver metastasis -A case report. Korean J Anesthesiol.

[3] Kawahara T, Hagiwara M, Takahashi H, Tanaka M, Imai K, Sawada J (2017). Cerebral Infarction by Paradoxical Gas Embolism During Laparoscopic Liver Resection with Injury of the Hepatic Vessels in a Patient without a Right-to-Left Systemic Shunt. Am J Case Rep.

[4] Bourdel N, Matsuzaki S, Bazin J-E, Pouly J-L, Mage G, Canis M (2007). Peritoneal tissue-oxygen tension during a carbon dioxide pneumoperitoneum in a mouse laparoscopic model with controlled respiratory support. Hum Reprod.

[5] Matsuzaki S, Bourdel N, Darcha C, Déchelotte PJ, Bazin J-E, Pouly J-L (2009). Molecular mechanisms underlying postoperative peritoneal tumor dissemination may differ between a laparotomy and carbon dioxide pneumoperitoneum: a syngeneic mouse model with controlled respiratory support. Surg Endosc.

[6] Matsuzaki S, Jardon K, Maleysson E, D’Arpiany F, Canis M, Bazin J-E (2010). Carbon dioxide pneumoperitoneum, intraperitoneal pressure, and peritoneal tissue hypoxia: a mouse study with controlled respiratory support. Surg Endosc.

[7] Sánchez-Margallo FM, Moyano-Cuevas JL, Latorre R, Maestre J, Correa L, Pagador JB (2011). Anatomical changes due to pneumoperitoneum analyzed by MRI: an experimental study in pigs. Surg Radiol Anat.

[8] Bazin JE, Gillart T, Rasson P, Conio N, Aigouy L, Schoeffler P (1997). Haemodynamic conditions enhancing gas embolism after venous injury during laparoscopy: A study in pigs. Br J Anaesth.

[9] Eiriksson K, Fors D, Rubertsson S, Arvidsson D (2011). High intra-abdominal pressure during experimental laparoscopic liver resection reduces bleeding but increases the risk of gas embolism. Br J Surg.

[10] Eryilmaz HB, Memiş D, Sezer A, Inal MT. The effects of different insufflation pressures on liver functions assessed with LiMON on patients undergoing laparoscopic cholecystectomy. Sci World J. 2012;2012:172575. 10.1100/2012/172575 PMC3349322 22619616

[11] Neudecker J, Sauerland S, Neugebauer E, Bergamaschi R, Bonjer HJ, Cuschieri A (2002). The European Association for Endoscopic Surgery clinical practice guideline on the pneumoperitoneum for laparoscopic surgery. Surg Endosc.

[12] Özdemir-van Brunschot DMD, van Laarhoven KCJHM, Scheffer G-J, Pouwels S, Wever KE, Warlé MC (2016). What is the evidence for the use of low-pressure pneumoperitoneum? A systematic review. Surg Endosc.

[13] O’Sullivan DC, Micali S, Averch TD, Buffer S, Reyerson T, Schulam P (1998). Factors involved in gas embolism after laparoscopic injury to inferior vena cava. J Endourol.

[14] Tan CN, Fraser AG (2007). Transesophageal echocardiography and cardiovascular sources of embolism: implications for perioperative management. Anesthesiology..

[15] Arthur ME, Landolfo C, Wade M, Castresana MR (2009). Inferior vena cava diameter (IVCD) measured with transesophageal echocardiography (TEE) can be used to derive the central venous pressure (CVP) in anesthetized mechanically ventilated patients. Echocardiography..

[16] Mugloo MM, Malik S, Akhtar R (2017). Echocardiographic Inferior Vena Cava Measurement As An Alternative to Central Venous Pressure Measurement in Neonates. Indian J Pediatr.

[17] Markou N, Grigorakos L, Myrianthefs P, Boutzouka E, Rizos M, Evagelopoulou P (2004). Venous pressure measurements in the superior and inferior vena cava: the influence of intra-abdominal pressure. Hepatogastroenterology..

[18] Royse CF, Newman S, Chung F, Stygall J, McKay RE, Boldt J (2010). Development and feasibility of a scale to assess postoperative recovery: the post-operative quality recovery scale. Anesthesiology..

[19] Chan A-W, Tetzlaff JM, Gøtzsche PC, Altman DG, Mann H, Berlin JA (2013). SPIRIT 2013 explanation and elaboration: guidance for protocols of clinical trials. BMJ..

[20] Schmandra TC, Mierdl S, Bauer H, Gutt C, Hanisch E (2002). Transoesophageal echocardiography shows high risk of gas embolism during laparoscopic hepatic resection under carbon dioxide pneumoperitoneum. Br J Surg.

[21] Royse CF, Saager L, Whitlock R, Ou-Young J, Royse A, Vincent J (2017). Impact of Methylprednisolone on Postoperative Quality of Recovery and Delirium in the Steroids in Cardiac Surgery Trial: A Randomized, Double-blind, Placebo-controlled Substudy. Anesthesiology.

[22] Bowyer A, Jakobsson J, Ljungqvist O, Royse C (2014). A review of the scope and measurement of postoperative quality of recovery. Anaesthesia..

[23] Ryan T, Petrovic O, Dillon JC, Feigenbaum H, Conley MJ, Armstrong WF (1985). An echocardiographic index for separation of right ventricular volume and pressure overload. J Am Coll Cardiol.

[24] Dindo D, Demartines N, Clavien P-A (2004). Classification of surgical complications: a new proposal with evaluation in a cohort of 6336 patients and results of a survey. Ann Surg.

[25] Hong JY, Kim JY, Choi YD, Rha KH, Yoon SJ, Kil HK (2010). Incidence of venous gas embolism during robotic-assisted laparoscopic radical prostatectomy is lower than that during radical retropubic prostatectomy. Br J Anaesth.

[26] Derouin M, Couture P, Boudreault D, Girard D, Gravel D (1996). Detection of gas embolism by transesophageal echocardiography during laparoscopic cholecystectomy. Anesth Analg.

[27] Cuesta MA, Meijer S, Paul MA, de Brauw LM (1995). Limited laparoscopic liver resection of benign tumors guided by laparoscopic ultrasonography: report of two cases. Surg Laparosc Endosc.

[28] Tranchart H, Dagher I (2014). Laparoscopic liver resection: a review. J Visc Surg.

[29] Otsuka Y, Katagiri T, Ishii J, Maeda T, Kubota Y, Tamura A (2013). Gas embolism in laparoscopic hepatectomy: what is the optimal pneumoperitoneal pressure for laparoscopic major hepatectomy?. J Hepatobiliary Pancreat Sci.

[30] Schmandra TC, Mierdl S, Hollander D, Hanisch E, Gutt C (2004). Risk of gas embolism in hand-assisted versus total laparoscopic hepatic resection. Surg Technol Int.

[31] Hong Y, Xin Y, Yue F, Qi H, Jun C (2017). Randomized clinical trial comparing the effects of sevoflurane and propofol on carbon dioxide embolism during pneumoperitoneum in laparoscopic hepatectomy. Oncotarget..

[32] de Jong KIF, de Leeuw PW (2019). Venous carbon dioxide embolism during laparoscopic cholecystectomy a literature review. Eur J Intern Med.

[33] Lantz PE, Smith JD (1994). Fatal carbon dioxide embolism complicating attempted laparoscopic cholecystectomy--case report and literature review. J Forensic Sci.

[34] Hou W, Zhong J, Pan B, Huang J, Wang B, Sun Z, et al. Paradoxical carbon dioxide embolism during laparoscopic surgery without intracardiac right-to-left shunt: two case reports and a brief review of the literature. J Int Med Res. 2020;48:300060520933816. 10.1177/0300060520933816 PMC7418236 32776784

[35] Paquet C, Deschamps A, Denault AY, Couture P, Carrier M, Babin D (2008). Baseline regional cerebral oxygen saturation correlates with left ventricular systolic and diastolic function. J Cardiothorac Vasc Anesth.

[36] Jersenius U, Fors D, Rubertsson S, Arvidsson D (2007). Laparoscopic parenchymal division of the liver in a porcine model: comparison of the efficacy and safety of three different techniques. Surg Endosc.

[37] Huntington JT, Royall NA, Schmidt CR (2014). Minimizing blood loss during hepatectomy: a literature review. J Surg Oncol.

[38] Kobayashi S, Honda G, Kurata M, Tadano S, Sakamoto K, Okuda Y (2016). An Experimental Study on the Relationship Among Airway Pressure, Pneumoperitoneum Pressure, and Central Venous Pressure in Pure Laparoscopic Hepatectomy. Ann Surg.

[39] Jayaraman S, Khakhar A, Yang H, Bainbridge D, Quan D (2009). The association between central venous pressure, pneumoperitoneum, and venous carbon dioxide embolism in laparoscopic hepatectomy. Surg Endosc.

