# Supplementary material for: Gas embolism under standard versus low pneumoperitoneum pressure during laparoscopic liver resection (GASES): study protocol for a randomized controlled trial
Source: Trials. 2021 Nov 15;22:807. doi: 10.1186/s13063-021-05678-8 (PMC8591437; doi:10.1186/s13063-021-05678-8)
Supplement: Supplementary file 2 — Additional file 2. [file 13063_2021_5678_MOESM2_ESM.pdf]

不同气腹压力对于腹腔镜下肝脏肿瘤切除术中气栓风险影响的随机、对照临床研究

知情同意书

受试者须知页

方案名称：不同气腹压力对于腹腔镜下肝脏肿瘤切除术中气栓风险影响的随机、对照临床研究
知情同意书版本号：1.0　 2020 年 8 月 21 日
研究机构：复旦大学附属中山医院
主要研究者：钟静

尊敬的受试者：

您被邀请参加一项临床试验，该项研究由复旦大学附属中山医院提供支持。请仔细阅读本知情同意书并慎重做出是否参加本项研究的决定。参加这项研究完全是您自主的选择。作为受试者，您必须在加入临床研究前给出您的书面同意书。当您的研究医生或者研究人员和您讨论知情同意书的时候，您可以让他/她给您解释您看不明白的地方。我们鼓励您在做出参与此项研究的决定之前，和您的家人及朋友进行充分讨论。您有权拒绝参加本研究，也可随时退出研究，且不会受到处罚，也不会失去您应有的权利。若您正在参加别的研究，请告知您的研究医生或者研究人员。本研究的背景、目的、研究过程及其他重要信息如下：

一、　　研究背景

腹腔镜肝脏手术伴随的气体栓塞的风险较高。气体栓塞会伴有一系列心律失常。低气腹压能降低术后疼痛评分，具有潜在的肝肾损伤的保护作用。然而气腹压力与气栓的发生概率还未有定论。

目前，经食道心脏超声（Transesophageal Echocardiography,TEE）是最敏感的监测气体栓塞的手段，能监测到 0.02mL/kg 的微量气体，同时，由于 TEE 不影响术者操作、不破坏无菌区等特点，已成为麻醉医师术中确诊气体栓塞的首选监测手段。

腹腔镜肝脏切除术采用的低中心静脉压（central vein pressure，CVP）技术，其本质是通过降低下腔静脉张力，从而减少肝静脉压力，使分离肝组织周围的静脉血管后向压力降低，从而提供一个干净的术野。目前多采用中心静脉置管测压来反应下腔-肝静脉系统的压力，有研究认为下腔静脉直径与 CVP 相关性良好，但也有研究发现，中心静脉测压获得的 CVP 与肝血流参数的改变不存在相关性，此结果对以 CVP 为监测指标减少切肝时出血和气栓发生风险的措施提出了质疑。因此，本实验

还采用 TEE 测量下腔静脉塌陷率（Inferior vena cava collapse rate，IVCCR）和直径来反应下腔静脉张力，探讨 IVCCR 和 CVP 的相关性。IVCCR=（呼气末 IVC 内径-吸气末 IVC 内径）/呼气末 IVC 内径。

二、 研究目的

本研究旨在探讨不同气腹压力对于腹腔镜肝脏肿瘤切除术中气栓风险和术后恢复情况的影响，以及 IVCCR 和 CVP 的相关性，为制定肝脏肿瘤腹腔镜手术治疗的最适气腹压力提供参考依据。

三、研究过程

1. 多少人将参与这项研究？

本项目为一项复旦大学附属中山医院主持的随机对照临床试验，共计有 140 名患者参与。

2. 研究步骤

如果您同意参加本研究，请您签署这份知情同意书。

① 在您入选研究前，医生将询问、记录您的病史，以确定您是否适合本次研究，不增加进行该次医疗活动以外的检查。

② 确定您可以参加本研究后，您将被随机分为 10mmhg 气腹压力组和 15mmhg 气腹压力组，您和手术医师对此均无影响。在手术前，研究医生会收集你的病史信息，进行体格检查和抽血。试验过程中进行有创血压、心电图、氧饱和度、中心静脉压，麻醉深度和肌松监测等监测技术来保障手术过程中的安全。我们将记录术中生命体征数据、麻醉用药情况、右心系统气栓分级、术中 CVP、术中 IVCCR、术中与气栓相关的并发症、术中血气分析、手术医生满意度。术后 40 分钟在麻醉后监护病房将再次采集血液，最后一次采集于术后第一天。手术后第一天，将会对您进行面谈和体格检查，手术后第 5 天或出院前再重复一次。您将在术后 30 天和 90 天接受电话采访，了解您目前的身体状况和术后并发症。

③ 您的出院时间不会因参与该研究而延误。出院后不作进一步检查。

3. 这项研究会持续多久？

在您住院期间的手术当天、术后第一天、术后第 5 天或出院前，会对您进行随访收集恢复情况的信息，在术后 1 个月和 3 个月时会电话随访满意程度以及并发症的情况

您可以在任何时间选择退出研究而不会失去您本应获得的任何利益。然而，如果在研究途中您决定退出本研究，我们鼓励您先和您的医生商议。如果您出现严重的不良事件，或者您的研究医生觉得继续参加研究不符合您的最佳利益，他/她会决定让您退出研究。申办者或者监管机构也可能在研究期间终止研究。但您的退出不

会影响您的正常医疗待遇与权益不受影响。

如果您因为任何原因从研究中退出，您可能被询问有关您参加研究的情况。如果医生认为需要，您也可能被要求进行实验室检查和体格检查。

4. 研究中收集的信息和生物标本

该研究收集的病人信息采用纸张及电子表格进行保存，由研究负责人保管，遵守保密原则，不泄露患者的相关信息。本次研究采集生物标本（血液）于检验后即刻销毁，无生物标本送出复旦大学附属中山医院。

四、风险与受益

1. 参加本研究的风险是什么？

本研究两种气腹压力都是目前实践操作中可安全使用的，因此原则上不会带来风险。然而，可能存在信息安全方面的风险。我们会尽全力保护您提供的信息不被泄露，在法律允许的范围内，尽一切努力保护您个人医疗资料的隐私。

本研究中我们所问您的一些问题可能会让您感到不舒服，你可以拒绝回答此类问题，同时，研究过程中您随时都可以休息。在研究中任何时刻，您都可以退出本研究。

如果在研究期间您出现任何不适，或病情发生新的变化，或任何意外情况，不管是否与研究有关，均应及时通知您的医生，他/她将对此作出判断并给与适当的医疗处理。

您在研究期间需要按时到医院随访，做一些检查，这将会占用您的一些时间，也可能给您造成麻烦或带来不方便。

2. 参加研究有什么受益？

实际受益：参与研究的患者可以从手术期间和术后延长的监测中获益。

潜在受益：如果受试者入选低气腹压力组，该方案可能降低术后疼痛评分，具有潜在的肝肾损伤的保护作用。更为重要的是，我们希望从您参与的本研究中得到的信息在将来能够使您或与您病情相同的病人获益。

五、备选的治疗方案

除了参与本研究，您可以接受您的医生提供的常规治疗。

六、研究结果的使用和个人信息的保密

研究过程中，我们将收集您的病史信息、实验室和影像学检查结果、随访信息，为保证隐私我们将对您的部分信息进行编码，您的个人标识符（例如姓名、出生日期、地址）将被代码（唯一的患者编号）代替，以便任何人无法确定您的身份。所有数据保存在主要研究者手中，在您和其他受试者的理解和协助下，通过本项目研究的结果可能会在医学杂志上发表，但是我们会按照法律的要求为您的研究记录保密。研究受试者的个人信息将受到严格保密，除非应相关法律要求，您个人信息不

会被泄露。必要时，政府管理部门和医院伦理委员会及其它相关研究人员可以按规定查阅您的资料。

七、关于研究费用及相关补偿

1. 研究所用的药物/器械及相关检查费用

本次试验内容不会额外增加诊疗费用，但是您需要缴纳常规麻醉费用，对于您同时合并的其他疾病所需的常规治疗和检查，也不在免费的范围之内。

2. 参加研究的补偿

无额外补偿。

3. 发生损伤后的补偿/赔偿

如果确实发生与该项研究相关的损伤，您可以获得由本单位提供的免费治疗，或按中国有关法律进行补偿/赔偿。

八、受试者的权利和相关注意事项

1. 您的权利

在参加研究的整个过程中，您都是自愿的。如果您决定不参加本研究，也不会影响您应该得到的其他治疗。如果您决定参加，会要求您在这份书面知情同意书上签字。您有权在试验的任何阶段随时退出试验而不会遭到歧视或受到不公平的待遇，您相应医疗待遇与权益不受影响。

2. 注意事项

作为受试者，您需要提供有关自身病史和当前身体状况的真实情况；告诉研究医生自己在本次研究期间所发现的任何不适；不得服用医生已告知的受限制药物、食物等；告诉研究医生自己最近是否 参与其他研究，或目前正参与其他研究。

九、获知信息的相关联系方式

如果在研究过程中有任何重要的新信息，可能影响您继续参加研究的意愿时，您的医生将会及时通知您。如果您对自己的研究数据，或研究结束后您希望知道本研究的发现。您可以在任何时间提出有关本项研究的任何问题，并得到相应的解答，请通过电话 13818185296 与 钟静 联系。

伦理委员会已经审查通过该研究，如果您有与自身权利/权益相关的任何问题，或者您想反映参与本研究过程中遭遇的困难、不满和忧虑，或者想提供与本研究有关的意见和建议，请联系复旦大学附属中山医院伦理委员会，联系电话：021-31587871，电子邮件：ec@zs-hospital.sh.cn。

受试者签字页

知情同意声明：

我已被告知此项研究的目的、背景、过程、风险及获益等情况。我有足够的时间和机会进行提问，问题的答复我很满意。

我也被告知，当我有问题、想反映困难、顾虑、对研究的建议，或想进一步获得信息，或为研究提供帮助时，应当与谁联系。

我已经阅读这份知情同意书，并且同意参加本研究。

我知道我可以选择不参加此项研究，或在研究期间的任何时候无需任何理由退出本研究。

我已知道如果我的状况更差了，或者我出现严重的不良事件，或者我的研究医生觉得继续参加研究不符合我的最佳利益，他/她会决定让我退出研究。无需征得我的同意，资助方或者监管机构也可能在研究期间终止研究。如果发生该情况，医生将及时通知我，研究医生也会与我讨论我的其他选择。

我将得到这份知情同意书的副本，上面包含我和研究者的签名。

受试者签名：____________________　　　　日期：___________

（注：如果受试者无行为能力/限制行为能力时，则需法定代理人签名和签署日期）

法定代理人签字：________________　　　　日期：___________

（注：如果受试者不能阅读该知情同意书时，则需一名独立见证人证明研究者已将知情同意书的所有内容告知了受试者，独立见证人需签名和签署日期）

独立见证人签字：________________　　　　日期：___________

研究者签名：____________________　　　　日期：___________

GASES-Patient information

Gas embolism under standard versus low pneumoperitoneum pressure during laparoscopic liver resection (GASES): study protocol for a randomized, controlled trial

Dear patient,

you are planned for a surgical procedure with general anesthesia, a procedure which requires mechanical ventilation.

Background of the study

Laparoscopic surgery needs intra-abdominal CO_2 influx to lift abdominal wall, as called the pneumoperitoneum, for the creation of a satisfied surgical space. Laparoscopic liver resection (LLR) may be correlated to a higher incidence of gas embolism. Patients will experience a series of arrhythmia correlated with the gas embolism. Therefore, a low level of 6-8mmhg pneumoperitoneum pressure (PP) is recommended in laparoscopic surgery according to recent studies. Low PP maintained during LLR improved postoperative pain and potentially decreased liver and kidney injury. However, the association between PP and air embolism has not been determined.

The low central venous pressure (CVP) technique more frequently used in LLR essentially reduces the hepatic venous pressure by reducing the inferior vena cava (IVC) tension, thereby reducing the posterior pressure of the separated veins around the liver tissue and thus providing a clear surgery field. Central venous catheterization is commonly used to measure CVP, which reflects the pressure of the IVC system. However, there is a doubt on the correlation between CVP and hepatic blood flow parameter, questioning the value of monitoring CVP to reduce the risk of hemorrhage and thrombus during liver resection.

So far TEE is the most sensitive method for monitoring gas embolism and capable of detecting 0.02mL/kg of gas. TEE could be an effective measurement to explore the correlation between IVC dilated rate (IVCDR) and CVP, and has gained increasing priority when diagnosing the intraoperative gas embolism among anesthesiologists because of its advantages such as avoiding influencing the operation of the surgeon and contaminating the aseptic area.

In view of these facts, we design the standard versus low PP during elective LLR trial. We hypothesize that a low PP (10 mmHg) maintained in controlled-low-CVP elective LLR will

lead to less gas embolism, therefore promotes outcome and reduces postoperative organ dysfunction and prolong survival, as compared to standard PP (15 mmHg).

Study description

In a first step, an anesthetist will first determine whether you are suitable for this study by means of a questionnaire. Before the surgical procedure, the study physician will collect information on your medical history, perform a physical examination and blood sampling. During the operation, you will be performed the LLR with a standard or low PP for the entire procedure of surgery. The PP that is maintained during LLR for you will be decided by a random lottery, which doctors and patients will have no influence on. Another blood sample will be taken 40 minutes after your recovery from anesthesia in the post-anesthesia care unit, and the last blood sample will be taken on the first day after the operation. On the first day after the operation, a patient interview and a physical examination, which are repeated on the third day after the operation. You will receive an interview on phone call about your current physical situation and postoperative complications at 30-day and 90-day after the operation.

Your discharge from the hospital is not delayed due to participation in the study. No additional examinations after hospital discharge are made.

Individual benefits, risks and burdens for the patient

The probability that you will be assigned to the group with the standard PP or the group with the lower PP is equal, 50% for both cases. A particular advantage for all patients participating in this study is that they can benefit from extended monitoring during and after the operation. Nevertheless, there are different risks and benefits for both groups.

If you are assigned to the standard PP group, you will be performed the operation maintaining an intra-abdominal pressure that is preferred by most of the surgeons all over the world. During standard PP, the airway pressure will be elevated and a series of cardiopulmonary changes will develop. A particular advantage of this PP level is that the surgical space is somewhat guranteed.

During lower PP, the influence of intra-abdominal is controlled and the cardiopulmonary function is not impaired, which is likley to help maintain a more stable circulation. However, the surgical space will be compromised and influence the surgical course. The compromised surgical space can be promoted in our trial by deep neuromuscular block with the standard

pump of cisatracurium. If the surgical space still can't be reversed, the PP will be transformed to the standard group.

Physical examination does not involve invasive or painful procedures. The study-related blood samples are, as far as possible, linked to the routine blood collection or carried out via existing vascular access. In the case of study-related venipuncture, there is a risk of infections, hematoms and nerve damage in the area of the puncture site or its surroundings. In the blood samples, laboratory tests are carried out, which allow a statement on the function of different organs, including the lungs. Blood samples will be destroyed immediately after the biomarker data is recorded.

Privacy policy

The data collected in the study will be saved both electronically and in paper form for at least 10 years in the coordination center for clinical trials in Shanghai and partly also in the Department of Anesthesia of the Zhongshan Hospital Fudan University in China (Director: Prof. Miao). This time period can be changed by new laws of China. Data access is granted to investigators of the study in Shanghai, whereby all parties must comply with the China data safety and privacy regulations. For this case your consent is required. The data will be processed by special software and statistically evaluated. In data processing, the identity of the patients is encrypted by means of a number code (pseudonymization). This code can be passed on to the relevant monitoring authorities. For inspection purposes the access to the encrypted data can be granted, but the person entitled to access is obliged to maintain secrecy. It is planned to publish the study results in one or more scientific journals. In this case, due to the pseudonymization conclusions on individual patients will not be possible. You have the right to access and to correct your data collected in the study. Informing your family practitioner about participation in the study is desirable, but requires your explicit consent.

Voluntariness and consent withdrawal

Participation in the study is on a voluntary basis and the rejection does not bring any disadvantages for further treatment of the patient. Data which may have been collected up to the date of consent withdrawal shall not be passed on, even in an anonymous form, and will not be included in the evaluation. The consent you have given may be revoked at any time without giving reasons!

The relevant contact information

If there is any significant new information during the course of the study that may affect your willingness to continue to participate in the study, your physician will inform you immediately. If you would like to know the findings of this study, you can put forward any questions about this study at any time and receive the germane answers. Please contact Zhong Jing at 13818185296.

Ethics committee will review through the study, if you have any questions related to their rights and interests, or you want to reflect the difficulties, discontent, and concerns in the process of participating in this study, or if you want to provide opinions and suggestions related to this study, please contact the Institutional Review Board of Zhongshan Hospital Fudan University(Phone number: 021-31587871, E-mail: ec@zs-hospital.sh.cn).

Informed Consent Statement:

I have been informed of the purpose, background, process, risks and benefits of this research. I had plenty of time and opportunity to ask questions, and I was satisfied with the answers.

I was also told who to contact when I had questions, wanted to report difficulties, concerns, suggestions for research, or wanted to get further information, or to help with research.

I have read this informed consent and agree to participate in this study.

I understand that I may opt out of the study or withdraw from the study at any time during the study without any reason.

I already know that if my condition gets worse, or if I have a serious adverse event, or if my study physician decides that it is not in my best interest to continue in the study, he or she will decide to withdraw me from the study. The sponsor or regulatory authority may also terminate the study during the study period without my consent. If this happens, the doctor will inform me immediately and the doctor will discuss with me for other options.

I will get a copy of this informed consent with my signature and that of the researcher.

______________________ Location, Date	______________________ Name, first name of the participating person
	______________________ Signature of the participating person
______________________ Location, Date	______________________ Name, first name of the participating person
	______________________ Signature of the legal representative of the participating person
______________________ Location, Date	______________________ Signature of the investigator
