# Supplementary material for: Gas embolism under standard versus low pneumoperitoneum pressure during laparoscopic liver resection (GASES): study protocol for a randomized controlled trial
Source: Trials. 2021 Nov 15;22:807. doi: 10.1186/s13063-021-05678-8 (PMC8591437; doi:10.1186/s13063-021-05678-8)
Supplement: Supplementary file 3 — Additional file 3. [file 13063_2021_5678_MOESM3_ESM.pdf]

Post-Operative Quality of Recovery Scale (PQRS)*

Physiological Factors

P1 Blood Pressure
Please record the patient's systolic blood pressure — 3= 90-140; 2=70-89 or 141-180; 1= <70 or >180

P2 Heart Rate
Please record the patient's heart rate — 3= 45-100; 2= 35-44 or 101-139; 1= <35 or >140

P3 Temperature — *Method 1. Sublingual 2. Tympanic 3. Other*
Please record the patient's temperature — 3=36-37.6; 2=35-35.9 or 37.7-38.9; 1=<35 or >39

P4 Respiration
Please record the patient's respiratory rate — 3= 10-20; 2= 5-9 or 21-30; 1=<5 or >30

P5 Oxygen use to maintain Spo_2
Please record oxygen requirement
3. Oxygen administered by protocol or not required
2. Any SpO_2 <95% requiring oxygen as an intervention
1. Any SpO_2 <90% requiring oxygen as an intervention

P6 Airway
Please record the number corresponding to the actual assessment
3. Self-maintenance of airway
2. Maintenance of airway with support
1. Device *in situ*

P7 Agitation
Please record the number corresponding to the actual assessment
3. Shows no sign of agitation
2. Patient shows occasional agitation
1. Patient shows severe agitation

P8 Consciousness
Please record the number corresponding to the actual assessment
3. Fully awake
2. Rousable on auditory or physical stimulation
1. Not rousable

P9 **Please touch your nose or please lift your head?**
Please record the number corresponding to the actual assessment
3. Patient follows command completely
2. Patient responds purposely but is unable to complete request
1. No patient response or purposeless movement

Nociceptive Factors

N1 **I am going to show you a series of faces and I would like you to indicate which face, number, or description most accurately describes your level of pain at the moment.**
Please show the appropriate face chart to the patient and record the number corresponding to the actual response (1 - 5).
If the patient is unable to sit-up to read the chart please read out the responses to the patient by saying, **I would like you to tell me which of the following descriptions of level of pain best describes how you are feeling at the moment.** Read out the text on the chart and record response (1-5).

N2 **I am going to show you a series of faces and I would you to indicate which face, number, or description most accurately describes your level of feeling nauseous or vomiting at the moment.**
Please show the appropriate face chart to the patient and record the number corresponding to the actual response (1 - 5).
If the patient is unable to sit-up to read the chart please read out the responses to the patient by saying, **I would like you to tell me which of the following descriptions of level of nausea or vomiting best describes how you are feeling at the moment.** Read out the text on the chart and record response (1-5).

Emotional Factors
E1 **I am going to show you a series of faces and I would like you to indicate which face, number, or description most accurately describes to what extent you feel sad, low, or depressed at the moment.**<br>Please show the appropriate face chart to the patient and record the number corresponding to the actual response (1 - 5).<br>If the patient is unable to sit-up to read the chart please read out the responses to the patient by saying, **I would like you to tell me which of the following descriptions of extent of sadness or depressed mood best describes how you are feeling at the moment.** Read out the text on the chart and record response (1-5).
E2 **I am going to show you a series of faces and I would like you to indicate which face, number, or description most accurately describes to what extent you feel anxious or nervous at the moment.**<br>Please show the appropriate face chart to the patient and record the number corresponding to the actual response (1 - 5).<br>If the patient is unable to sit-up to read the chart please read out the responses to the patient by saying, **I would like you to tell me which of the following descriptions of extent of anxiousness or nervousness best describes how you are feeling at the moment.** Read out the text on the chart and record response (1-5).
Cognitive Factors
C1 **Please tell me your name, the city we are in and your date of birth.**<br>Please record the number of correct responses (1 – 3)
C2 **I am going to read you a list of numbers. Listen carefully, then when I am finished, I would like you to repeat them back to me in the same order that I read them. So, for example, if I said 1,2,3, you would say 1,2,3.**<br>Read out the digits given at the rate of one per second. Stop after failure at any point. Please record the item number of the last line correctly recalled.<br>1 **6, 7**<br>2 **2, 5, 3**<br>3 **6, 3, 8, 2**<br>4 **5, 7, 3, 6, 1**<br>5 **4, 3, 9, 5, 2, 8**<br>6 **1, 7, 9, 5, 3, 2, 4**
C3 **I am going to read you some more numbers, but this time when I stop I would like you to say them in reverse order. So, for example, if I said 1,2,3 you would say 3,2,1.**<br>Read out the digits given at the rate of one per second. Stop after failure at any point. Please record the item number of the last line correctly recalled.<br>1 **5, 6**<br>2 **3, 7, 4**<br>3 **5, 9, 1, 3**<br>4 **7, 6, 8, 2, 4**<br>5 **3, 6, 1, 5, 9, 2**<br>6 **1, 4, 8, 6, 3, 9, 2**
C4 **I am going to read out a list of words. Please listen carefully as when I have finished I would like you to repeat back to me as many of the words as you can remember. You can say them in any order and if you are not sure if you have said a word, say it just in case.**<br>Read the words to the patient at about 1 per second. Please record the number of correct responses.<br>DRUM, CURTAIN, BELL, COFFEE, SCHOOL, PARENT, MOON, GARDEN, HAT, FARMER, NOSE, TURKEY, COLOR, HOUSE, RIVER
C5 **I am going to name a letter and I would like you to state as many words as you can in 30 secsonds that begin with this letter, try to avoid proper nouns, such as peoples' names, names of countries etc, numbers or the same word with a different ending such as long, longer, longish. The letter is "C."** Time for 30 seconds using a stopwatch and stop patient at this time point.<br>Please record the number of words correctly given in the 30 second time period.

*Extract from: Royse CF, Newman S, Chung F, Stygall J, McKay RE, Boldt J, et al. Development and feasibility of a scale to assess postoperative recovery: the post-operative quality recovery scale. Anesthesiology. 2010 Oct;113(4):892–905.

术后恢复质量量表

生命体征(Physiological Factors)			
P1 收缩压 mmHg	☐ 3 分(90-140)	☐ 2 分(70-89 或 141-180)	☐ 1 分(<70 或>180)
P2 心率 次/分	☐ 3 分(45-100)	☐ 2 分(35-44 或 101-139)	☐ 1 分(<35 或>140)
P3 体温 C°	☐ 3 分(36-37)	☐ 2 分(35-35.9 或 37.7-38.9)	☐ 1 分(<35 或>39)
P4 呼吸 次/分	☐ 3 分(10-20)	☐ 2 分(5-9 或 21-30)	☐ 1 分(<5 或>30)
P5 氧饱和度 %	☐ 3 分(>95)	☐ 2 分(90-95)	☐ 1 分(<90)
P6 气道	☐ 3 分(自主)	☐ 2 分(辅助支持)	☐ 1 分(原位设备)
P7 躁动	☐ 3 分(无躁动)	☐ 2 分(偶尔躁动)	☐ 1 分(严重躁动)
P8 觉醒	☐ 3 分(完全清醒)	☐ 2 分(对听觉或刺激有反应)	☐ 1 分(无法唤醒)
P9 指令反应	☐ 3 分(完全)	☐ 2 分(能听懂无法完成)	☐ 1 分(无)

疼痛反应(Nociceptive Factors)					
N1 很痛？	☐ 5 分(非常同意)	☐ 4 分(同意)	☐ 3 分(不确定)	☐ 2 分(不同意)	☐ 1 分(非常不同意)
N2 恶心呕吐？	☐ 5 分(非常同意)	☐ 4 分(同意)	☐ 3 分(不确定)	☐ 2 分(不同意)	☐ 1 分(非常不同意)

日常活动(Activities of daily living)			
A1 站立	☐ 3 分(轻松)	☐ 2 分(有困难)	☐ 1 分(无法完成)
A2 行走	☐ 3 分(轻松)	☐ 2 分(有困难)	☐ 1 分(无法完成)
A3 吃喝	☐ 3 分(轻松)	☐ 2 分(有困难)	☐ 1 分(无法完成)
A4 穿衣	☐ 3 分(轻松)	☐ 2 分(有困难)	☐ 1 分(无法完成)

情绪表达(Emotional Factors)					
E1 悲伤失落抑郁？	☐ 5 分(非常同意)	☐ 4 分(同意)	☐ 3 分(不确定)	☐ 2 分(不同意)	☐ 1 分(非常不同意)
E2 紧张焦虑？	☐ 5 分(非常同意)	☐ 4 分(同意)	☐ 3 分(不确定)	☐ 2 分(不同意)	☐ 1 分(非常不同意)

认知功能(Cognitive Factors)

C1 请告诉我你的姓名、生日和我们现在何处。 ☐ 3 分(完全) ☐ 2 分(部分) ☐ 1 分(无)

C2 我将报一串数字，请按同样的顺序复述一遍。比如 我说 1,2,3，你说 1,2,3

☐ 1 分 6,7	☐ 2 分 2,5,3	☐ 3 分 6,3,8,2
☐ 4 分 5,7,3,6,1	☐ 5 分 4,3,9,5,2,8	☐ 6 分 1,7,9,5,3,2,4

C3 我将报一串数字，请按倒过来的顺序复述一遍。比如 我说 1,2,3，你说 3,2,1

☐ 1 分 5,6	☐ 2 分 3,7,4	☐ 3 分 5,9,1,3
☐ 4 分 7,6,8,2,4	☐ 5 分 3,6,1,5,9,2	☐ 6 分 1,4,8,6,3,9,2

C4 我将告诉你一串东西的名字，请尽可能多地告诉我你记得多少，不确定有没有可以说好像有。

锣鼓、窗帘、铃铛、咖啡、学校、父母、月亮、花园、帽子、农民、鼻子、母鸡、颜色、房屋、河流

☐ 1 分(0-2) ☐ 2 分(3-5) ☐ 3 分(6-8) ☐ 4 分(9-11) ☐ 5 分(12-14) ☐ 6 分(15-16)

C5 我将给你一个拼音字母，请 **30** 秒内尽可能多地说出以此为声母的字。比如，我说 C，你说 “长，刺，馋”

☐ 1 分(0-5) ☐ 2 分(6-10) ☐ 3 分(11-15) ☐ 4 分(16-20) ☐ 5 分(21-25) ☐ 6 分(26-30)

患者总体评价(Overall Patient Perspective)

O1 你觉得自己术后恢复得好吗？包括日常活动、思维清晰、工作能力、对手术麻醉过程满意等

☐ 5 分(非常同意) ☐ 4 分(同意) ☐ 3 分(不确定) ☐ 2 分(不同意) ☐ 1 分(非常不同意)
