# Supplementary material for: Gas embolism under standard versus low pneumoperitoneum pressure during laparoscopic liver resection (GASES): study protocol for a randomized controlled trial
Source: Trials. 2021 Nov 15;22:807. doi: 10.1186/s13063-021-05678-8 (PMC8591437; doi:10.1186/s13063-021-05678-8)
Supplement: Supplementary file 4 — Additional file 4. [file 13063_2021_5678_MOESM4_ESM.pdf]

伦理委员会批准函

Ethics Committee Approval Letter

审查编号 Approval No.: B2020-206R

项目名称 Study Title	不同气腹压力对于腹腔镜下肝脏肿瘤切除术中气栓风险影响的随机、对照临床研究		
试验产品名称 Study Product Name	NA	产品类别/规格 Product Category	NA
批准文号及发文单位(Approval No. and Issued By)<br>NA		研究分期(Phase of Study)<br>NA	
主要研究者(Principal Investigator)<br>钟静		申办者(Sponsor)<br>复旦大学附属中山医院	
审查方式及日期 (Type & Date of Review)	■会议审查 (Meeting Review) 2020 年 08 月 20 日<br>■快速审查(Expedited Review) 2020 年 08 月 21 日		
会议地点 (Meeting Location)	复旦大学附属中山医院 5 号楼 211 会议室		
会议出席情况 (Meeting Attendance)	出席(Attendance) 10 人，投票(Vote) 10 人，回避(Avoidance) 0 人		

下列研究相关文件已经批准

The following documents have been approved

1. 研究方案：v2.0 2020.08.20
2. 知情同意书：v2.0 2020.08.20
3. 研究者简历
4. 研究人员名单
5. 病例报告表：v1.0, 2020.08.08

审查决定 Decision for this proposal and have been [√]:

[√] 同意 Approval

持续审查频率： □3 个月/3 Months □6 个月/6 Months ■1 年/1Year □不适用/NA

批准函有效期至： 2021 年 08 月 21 日

主任委员/副主任委员签名 Chair/Vice Chair Signature: 秦新裕印

批准日期 Approval Date:

复旦大学附属中山医院伦理委员会(盖章)Stamp of ZSEC 复旦大学附属中山医院医学伦理委员会

2020.8.21

A-024 版本：3.4

Ethics Committee Approval Letter

Approval NO: B2020-206R

**Study Title**	Gas embolism under standard versus low pneumoperitoneum pressure during laparoscopic liver resection (GASES): a randomized, controlled trial		
**Study Product Name**	NA	**Product Category**	NA
**Approval NO. and Issued By:** NA		**Phase of Study:** NA	
**Principal Investigator**<br><br>Jing Zhong		**Sponsor**<br><br>Zhongshan Hospital Fudan University	
**Type & Date of Review**	■ **Meeting Review**: 20-08-2020<br><br>■ **Expedited Review**: 21-08-20		
**Meeting Location**	No.211 Meeting Room, Building 5, Zhongshan Hospital Fudan University		
**Meeting Attendance**	Attendance: 10 Persons<br><br>Vote: 10 Persons<br><br>Avoidance: 0 Person		
The following documents have been approved:<br><br>1. Study design: version 2.0 of 20-08-2020<br>2. Informed consent: version 2.0 of 20-08-2020<br>3. Principal investigator profile<br>4. The list of investigators<br>5. Case report form: version1.0 of 08-08-2020			
Decision for this proposal and have been [√]:<br>[√]Approval<br>Frequency of continuous review: □ 3Months □ 6Months ■1Year □ NA<br><br>Expiration of approval letter: 21-08-2021<br>Chair/Vice Chair Signature: Xinyu Qin<br><br>Approval Date: 21-08-2020 Stamp of ZSEC:			

A-024 Version:3.4
