# Supplementary material for: Gas embolism under standard versus low pneumoperitoneum pressure during laparoscopic liver resection (GASES): study protocol for a randomized controlled trial
Source: Trials. 2021 Nov 15;22:807. doi: 10.1186/s13063-021-05678-8 (PMC8591437; doi:10.1186/s13063-021-05678-8)
Supplement: Supplementary file 5 — Additional file 5: Appendix 1. Postoperative organ function [file 13063_2021_5678_MOESM5_ESM.docx]

Appendix 1. Postoperative organ function

| Supine position, upper body elevation 30–45°, oxygen saturation stable for 10 min under air condition? | Y🞎 N🞎 | | if Y | | | | SPO_2_ [%]: | | | |
| --- | --- | --- | --- | --- | --- | --- | --- | --- | --- | --- |
|  |  | | if N | | | | SPO_2_ [%]: | | FiO_2_ [%]: |  |
| RR [/min] | | | | | | | | | | |
| HR [/min] | | | | | MAP [mmHg] | | | | | |
| T [°C] | | | | ^Tympanic membrane^🞎 ^Armpit^🞎 ^Inguen^🞎 ^Mouth^🞎  ^Rectum^ 🞎 | | | | | | |
|  | | | | ^Other^ 🞎 | | | | Specification: | | |
| Airway secretion | Y🞎 N🞎 | if Y | | | | Purulent🞎 Non-purulent🞎 | | | | |
| VAS dyspnea [1–10 cm] | | | | | VAS static [1–10 cm] | | | | | |
|  | | | | | VAS motion [1–10 cm] | | | | | |
